# Internet-Based Mental Health Intervention for Depressive Symptoms in Young Adults: Cost-Effectiveness Analysis

**DOI:** 10.2196/68167

**Published:** 2025-12-15

**Authors:** Joyce H S You, Scotty W C Luk, Dilys Y W Chow, Arthur D P Mak, Winnie W S Mak

**Affiliations:** 1 School of Pharmacy, Faculty of Medicine, The Chinese University of Hong Kong, 8th Floor, Lo Kwee-Seong Integrated Biomedical Sciences Building, Area 39, Shatin, N.T., Hong Kong SAR, China, 852-3943-6830, 852-2603-5295; 2 University Medical Service Office, The Chinese University of Hong Kong, Hong Kong SAR, China; 3 Department of Psychiatry, Faculty of Medicine, The Chinese University of Hong Kong, Hong Kong SAR, China; 4 Department of Psychology, Faculty of Social Science, The Chinese University of Hong Kong, Hong Kong SAR, China

**Keywords:** internet, cognitive behavioral therapy, telehealth, depression, university students, health economic analysis

## Abstract

**Background:**

Internet-based cognitive behavioral therapy (CBT) provides psychological interventions to individuals with mild depressive symptoms.

**Objective:**

This study aimed to examine the potential cost-effectiveness of internet-based guided-CBT in university students with mild depressive symptoms from the perspective of service providers in Hong Kong.

**Methods:**

The outcomes of low-intensity guided internet-based CBT and in-person CBT in a hypothetical cohort of university students with mild depressive symptoms were examined using a 5-year decision-analytic model. Model inputs were obtained from published literature and local data. Model outcomes included direct medical cost, school dropouts, and quality-adjusted life years (QALYs). Sensitivity analyses were conducted on all model parameters.

**Results:**

Compared to the in-person group, the internet group gained higher QALYs by 0.0211 QALYs, lowered school dropouts by 0.052%, and saved US $249 in the base-case analysis. In one-way sensitivity analysis, the internet group gained higher QALYs at a lower cost than the in-person group throughout the variation of all model inputs. Probabilistic sensitivity analysis showed that the internet group was cost-effective (at willingness-to-pay threshold was US $48,119/QALY) in 99.7% of the 10,000 Monte Carlo simulations.

**Conclusions:**

Internet-based CBT appears to be the cost-effective option when compared to in-person CBT for university students with mild depressive symptoms from the perspective of service providers in Hong Kong.

## Introduction

Depression is a leading cause of disability worldwide. The World Health Organization (WHO) reported that approximately 33% first-year college students in 8 countries were screened positive for a common *DSM-IV* (*Diagnostic and Statistical Manual of Mental Disorders IV*) mental disorders [1]. The pooled depression prevalence in university students from 81 studies (near 1.5 million subjects) was estimated to be 34% [2].

Cognitive behavioral therapy (CBT) is a widely used psychological therapy for symptoms of depressive disorders and has been traditionally administered as in-person interventions. Young adults with mild symptoms of depression typically delay face-to-face intervention with a therapist, plausibly associated to several possible reasons. Perceived stigma and embarrassment at face-to-face intervention is one potential barrier [3]. Also, the accessibility of face-to-face psychological interventions may also be limited by monetary or time requirements, as well as by the service delivery barriers, such as insufficient psychological services and inadequate workforce numbers [3]. Internet-based CBT interventions provide alternative options that are self-paced and highly accessible, and computerized low-intensity CBT is recommended by the National Institute for Health and Care Excellence (NICE) as an option for mild depression [4]. Low-intensity CBT is a structured self-help program, including 6‐8 brief sessions (20‐30 min per session) over 9‐12 weeks [4]. Meta-analyses reported superior efficacy of internet-based CBT when compared to the waitlist or usual care [5,6]. Offering guided internet-based low-intensity CBT could enhance the treatment and health economic outcomes of mental health management for university students with mild symptoms of depression. In addition to clinical evidence, health economic findings are important evidence to facilitate the informed decision-making process for policy makers on the uptake of a health technology. This study aimed to examine the potential cost-effectiveness of internet-based guided CBT in university students with mild depressive symptoms from the perspective of service provider in Hong Kong.

## Methods

### Ethical Considerations

The protocol of retrospective review had been approved by the Joint Chinese University of Hong Kong–New Territories East Cluster Clinical Research Ethics Committee. The need for informed consent was waived by the Joint Chinese University of Hong Kong–New Territories east Cluster Clinical Research Ethics Committee. Data deidentification was performed in the retrospective data review process, and no compensation was provided.

### Model Overview

**Figure 1.**
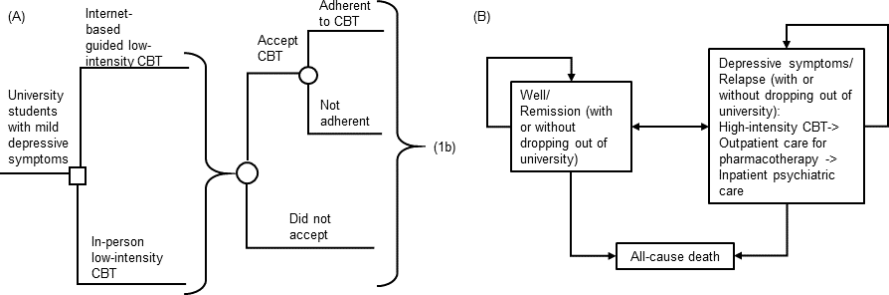
(A-B) Simplified decision-analytical model. Depressive symptoms/relapse status included cases of mild, moderate, or severe depressive symptoms. CBT: cognitive behavioral therapy.

A decision-analytic model (Figure 1A–B), including a short-term decision tree model followed by a 5-year Markov model, was designed to evaluate the potential health economic outcomes of a hypothetical cohort of university students with mild depressive symptoms managed by two strategies: (1) therapist-guided, internet-based low-intensity CBT (internet group) or (2) conventional in-person low-intensity CBT (in-person group). Depression symptom classifications adopted the Patient Health Questionnaire-9 (PHQ-9) [7]: mild (PHQ-9 score 5‐9), moderate (PHQ-9 score 10‐14), or severe (PHQ-9 score ≥15).

Low-intensity CBT (delivered in-person or via the internet guided by a therapist) recommended by NICE for mild depressive symptoms is a structured self-help program that includes 6‐8 sessions over 9‐12 weeks. In the in-person group, the participants work through the CBT-based self-help workbooks with brief support from the therapist in face-to-face sessions (20‐30 min per session). In the internet-based CBT, the low-intensity CBT program was offered via the internet platform. The participants work through the CBT-based self-help materials (resembling the structure of face-to-face CBT) delivered by online modules, and the therapist provided written feedback to the participant’s self-help work by emails or texting [4].

All hypothetical students with mild depressive symptoms entered a short-term model mirroring the duration of the low-intensity CBT program (Figure 1A). The hypothetical student (in both groups) might accept the CBT program, and those who accepted the therapy might adhere with the course of CBT. Upon the completion of low-intensity CBT, the student might recover well or deteriorate (showing moderate or severe depressive symptoms), and all were advanced to a 5-year Markov model (Figure 1B).

In the Markov model (Figure 1B), the hypothetical subjects proceeded through health states (Markov states) in each model cycle according to transition probabilities [8]. The present Markov model consisted of three key Markov states (well/remission, depressive symptoms/relapse, and all-cause death). Within the depressive symptoms/relapse status, cases are further differentiated (by proportion) into depressive symptoms of mild, moderate, or severe. The students who deteriorated or did not achieve remission with the low-intensity CBT program were stepped up to high-intensity CBT and outpatient psychiatric care for pharmacotherapy [4]. Students with severe depressive symptoms might be admitted to hospital for inpatient psychiatric care. In each model cycle, all hypothetical students (those in remission and those with depressive symptoms) might (or might not) permanently dropout from the university study. The model outcomes included direct medical cost, dropout, and quality-adjusted life years (QALYs). The model time horizon was 5 years, and the cycle length was 1 month. With the non-responding and recurring nature of depressive symptoms, a timeframe of 5 years was adequate to capture the outcomes of internet-based and conventional in-person low-intensity CBT, and the downstream costs and QALYs associated with deteriorated and non-responsive depressive symptoms.

### Clinical Inputs

The clinical model inputs (Table 1) were retrieved from published clinical studies. A MEDLINE search (2000‐2023) was performed using keywords such as “depressive disorders,” “internet,” “telehealth,” “telemedicine,” “cognitive behavioral therapy,”“cognitive behavioral therapy,” “pharmacotherapy,” “uptake,” “compliance,” “remission,” and “deterioration.” Selection criteria of published studies included: (1) Reports published in English language, (2) adults with symptoms of depressive symptoms, (3) event rates of remission and/or deterioration, and (4) individuals’ uptake of the delivery platform and (5) compliance of delivery platform (online and in-person) for CBT were reported. Meta-analyses and randomized controlled trials were the preferred types of studies. When multiple clinical randomized trials were available for the same model input, the base-case value was derived by the weighted average of the reported findings.

**Table 1.** Model input parameters.

Parameters	Base-case value	Range of sensitivity analysis	Distribution	Source of Information
Clinical inputs				
Low-intensity CBT^a^ for mild depressive symptoms, event rate (%)				
Recovery to well	63.9	51.1‐76.7	Beta^b^	[9]
Deterioration to moderate-severe symptoms	5.3	4.2‐6.4	Beta	[9]
Relative difference in effectiveness with internet-based versus in-person CBT, n	1.00	0.95‐1.05	Triangular	[10]
No intervention for mild symptoms, event rate (%)				
Recovery to well	25.0	20.0‐30.0	Beta	[11]
Deterioration to moderate-severe symptoms	9.1	7.3‐10.9	Beta	[12]
Acceptance of CBT, event rate (%)				
In-person	63.30	60.1‐66.5	Beta	[13]
Internet-based	75.3	71.5‐79.1	Beta	[13]
Compliance to CBT, event rate (%)				
In-person	83.9	75.7‐92.1	Beta	[14]
Internet-based	80.8	73.0‐88.7	Beta	[14]
Monthly probabilities for Markov modeling				
Age-specific all-cause mortality, event rate (%)	0.0025	0.0020‐0.0030	Beta	[15,16]
Relative risk of mortality with depressive symptoms, n	1.81	1.58‐2.07	Triangular	[17]
Remission, event rate (%)	0.54	0.43‐0.65	Beta	[18]
Relapse, event rate (%)	2.44	1.95‐2.93	Beta	[18]
Proportion of mild symptoms among relapse (mild-moderate-severe), event rate (%)	24.3	19.2‐28.8	Beta	[19]
Proportion of moderate symptoms among moderate-severe cases, event rate (%)	50	40‐60	Beta	[19]
Psychiatric hospitalization among severe cases, event rate (%)	1.78	1.42‐2.14	Beta	[20]
Dropout from university by mental health status, event rate (%)				[21]
Well/remission	0.124	0.099‐0.149	Beta	
Depressive symptoms	0.193	0.154‐0.232	Beta	
Utility inputs				
Age, years	21	20‐22	Triangular	Cohort of local university students
Age-specific utility, n	0.92	-	-	[22]
Remission, n	0.80	0.72‐0.88	Triangular	[23]
Mild depressive symptoms, n	0.62	0.56‐0.68	Triangular	[23]
Moderate depressive symptoms, n	0.48	0.43‐0.53	Triangular	[23]
Severe depressive symptoms, n	0.33	0.30‐0.36	Triangular	[23,24]
Cost inputs				
Low-intensity CBT for depressive symptoms				[4]
Number of sessions	7	6‐8	Triangular	
Duration of each session (min)	30	-	-	
High-intensity CBT for depressive symptoms				
Number of sessions	18	16‐20	Triangular	[4]
Duration of each session (minutes)	60	-	-	
Psychotherapist service per hour (US $)	54	43‐62	Triangular	Cohort of local university students
Platform overhead for internet-based CBT (US $)	24	20‐29	Triangular	[25]
Percentage of time spent by psychotherapist in guided interned-based CBT (compared to in-person CBT)	9.23	7.38‐11.1	Beta	[26]
Psychiatric outpatient (US $ per patient-month)	231	160‐335	Gamma	Cohort of local university students
Psychiatric hospitalization (US $ per episode)	1500	900‐2100	Gamma	[27,28]

a CBT: cognitive behavioral therapy.

b Parameter-specific distribution used in probabilistic sensitivity analysis.

The effectiveness of low-intensity CBT for mild depressive symptoms was retrieved from the findings of a prospective, pre- and post-treatment study of low-intensity CBT; they reported the recovery (63.9%) and deterioration (5.3%) rates for individual with mild common mental disorders (depressive disorders and anxiety disorders) in Hong Kong [9]. A systematic review (including 17 studies) reported that electronically delivered CBT was more effective than face-to-face CBT at lowering severity of depression symptoms and concluded that electronically delivered CBT was at least as effective as in-person CBT [10]. The relative difference in the effectiveness of CBT delivered via internet versus in-person was therefore assumed to be 1.00 in the present model. A recent Canada health technology assessment study on the cost-effectiveness of internet-delivered CBT for major depression had applied a spontaneous recovery rate of 25% for individuals who did not receive any treatment [11], and the present model adopted the spontaneous recovery rate for individuals with no intervention (who did not accept or were non-adherent to the CBT) in both study arms. The occurrence rate of deterioration in the individuals with no interventions was obtained from an individual participant data meta-analysis on internet-delivered CBT (including 16 trials with total 3805 participants), reporting that clinically significant deterioration had occurred in 9.1% participants in the control groups [12]. The preference of university students for digital technology versus in-person interventions when encountered mental health needs was examined in a survey study (n=572), and the likelihood of seeking help through online and in-person methods was 75.4%% and 63.3%, respectively [13]. The compliance to CBT was retrieved from the findings of a meta-analysis on adherence to internet-based (80.8%) and face-to-face (83.9%) CBT for depression (including 24 trials) [14].

The monthly probability of mortality in the subjects with depressive symptoms was approximated by all-cause mortality from age-specific mortality [15,16] and relative risk of mortality in individuals with depression (1.18) reported by a meta-analysis (including 25 studies and n=106,628) on excess mortality associated with depression [17]. The monthly probabilities of relapse from well (2.44%) and remission from depressive symptoms (0.54%) after the completion of low-intensity CBT in both study arms were estimated from the findings of a long-term follow-up study of depression in primary care setting over 3‐5 years (n=248; median 46 months, IQR 43-51 months) [18]. Of the relapsed cases from those who recovered well, the proportion of relapsed cases with mild symptoms (24.3%) and the proportion of relapsed cases with moderate symptoms (50%) among the relapsed cases with moderate-to-severe symptoms were retrieved from a retrospective cohort study (n=687) of the recurrence among patients with a history of major depressive disorder [19]. The monthly probability of psychiatric hospitalization (1.78%) among those with severe depressive symptoms was estimated from the 12-month psychiatric hospitalization rate reported by a retrospective database analysis (n=248) on hospital utilization in patients with major depressive disorder [20]. The monthly probabilities of school dropout among students with remission (0.124%) and those with depressive symptoms (0.193%) were approximated from the findings of a long-term follow-up study (for 4.8 years) on mental health and school dropout in over 3000 individuals aged 16‐29 years [21].

### Utility Inputs

The QALYs gained by each hypothetical student was calculated by the time spent in various health states, adjusting by the health state-specific utility (Table 1). The QALYs gained were discounted to the current year using a 3% annual discounting rate. A study reported the age-specific scores for quality of life from a US population-based survey [22], and we adopted the US age-specific score as the utility value in the present model. The utility values of remission and mild-to-severe symptoms of depression were adopted the corresponding values applied in cost-effectiveness analyses on the management of depression [23,24].

### Cost Inputs

The cost analysis was conducted from the perspective of service providers in Hong Kong. The direct cost items (Table 1) were costs for in-person low-intensity CBT, internet-supported guided low-intensity CBT, in-person high-intensity CBT, and costs of psychiatric outpatient and inpatient treatment for depressive symptoms. The cost of in-person CBTs were estimated by the number of sessions (per intensity level) [4] and the local hourly wages of the psychotherapist. The overhead for internet-supported CBT in our model adopted the overhead cost of an online-guided CBT program for depression examined in a randomized controlled study [25]. The percentage of the psychotherapist’s time (9.23%) for guided CBT versus in-person CBT was retrieved from the report of randomized controlled trials of internet-delivered versus in-person CBT for depression [26]. The cost per psychiatric hospitalization was approximated by the length of hospital stay for depression and cost per day of psychiatric hospitalization [27,28]. We conducted a retrospective review of psychiatric resource utilization in a cohort of local university students who received ambulatory psychiatric care for depressive symptoms. A cohort of students of the Chinese University of Hong Kong who had been referred by the University Health Services in 2017‐2020 to outpatient psychiatry care for depression symptoms (n=100; 32% men, median age 21 [IQR 20‐22] years) were reviewed on the psychiatric care reimbursement claims. The investigators had no access to information that could identify individual students during or after data collection. The median cost per patient month (US $231; 25th and 75th percentile US $160 and US $335) were applied as psychiatric outpatient costs (per patient month) for depressive symptoms. The costs were adjusted to the current year by an annual discounting rate of 3%.

### Data Analysis

The expected cost and QALY of both study arms were generated in the base-case analysis. A CBT strategy was the dominant strategy if it gained higher QALYs at a lower cost than the comparator. When a strategy gained higher QALYs at a higher cost than the comparator, incremental cost per additional QALY gained (ICER) was calculated as follows: Δcost/ΔQALYs. The WHO recommended that ICER less than 1×gross domestic product (GDP) per capita to be highly cost-effective [29]. The 2023 GDP per capita of Hong Kong (US $48,119) was adopted as the willingness-to-pay (WTP) threshold (cost per additional QALY gained) in the present analysis [30]. A CBT strategy was accepted as cost-effective if (1) it was the dominant option (the strategy was less costly and more effective), or (2) the strategy was more costly and more effective, and its ICER versus the comparator was less than the WTP threshold.

The robustness of the base-case results was examined by sensitivity analysis. One-way sensitivity analysis was conducted for all model inputs. The range for sensitivity analysis of each model input was either the 95% CI range, high/low values of the variable, interquartile range, standard deviation, or (if above were not reported) varied by 20% of the base-case value. Probabilistic sensitivity analysis examined the impact of simultaneous variation of all model inputs. The cost and QALYs were generated for 10,000 times using the Monte Carlo simulation by randomly drawing each model input from the parameter-specific distribution (Table 1). The incremental cost and incremental QALYs of the internet group were presented in a scatter plot. All analyses were performed using TreeAge Pro 2023 (TreeAge Software Inc) and Excel 2016 (Microsoft Corporation).

## Results

The internet group gained higher QALYs (by 0.0211 QALYs) and lowered dropout rate (by 0.0528%) at a reduced cost (US $249 saved) versus the in-person group in the base-case analysis (Table 2), and therefore was the (dominant) cost-effective option at ICER −11,801 US $/QALY.

**Table 2.** Base-case analysis results.

	Costs (US $)	Incremental cost (US $)	School dropout	Incremental Dropout	QALYs^a^	Incremental QALYs
Internet group	8498	−249	9.8645%	−0.0528%	2.5627	0.0211
In-person group	8747	-	9.9174%		2.5416	-

a QALYs: quality-adjusted life-years.

**Figure 2.**
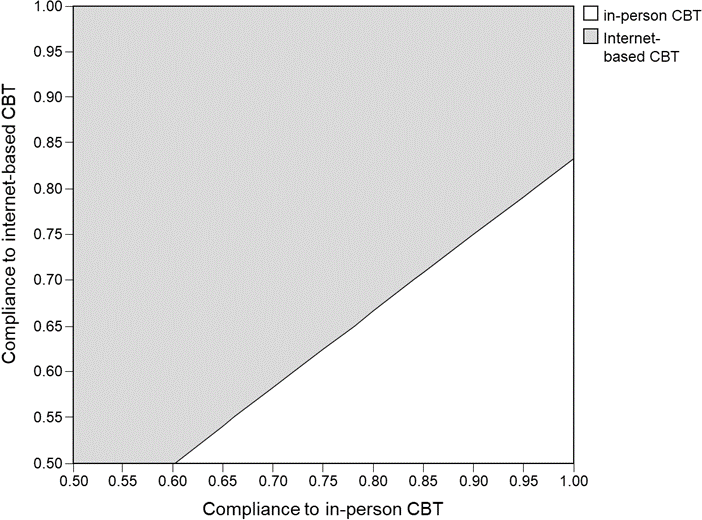
Two-way sensitivity analysis of compliance to internet-based and in-person CBT. Gray zone: Combinations of parameters led to the internet group as the cost-effective option. White zone: Combinations of parameters led to the in-person group as the cost-effective option. CBT: cognitive behavioral therapy.

In one-way sensitivity analysis, the internet-based group remained to gain higher QALYs at lower costs than the in-person group throughout the variation of all model inputs, and no threshold value was identified. The compliance rates to internet-based CBT and in-person CBT were the most influential factors on the ICER of internet-based CBT. When the compliance rate to internet-based CBT varied over the range of sensitivity analysis (from 73.0% to 88.7%), the ICER reduced from −10,116 USD/QALY to −23,892 USD/QALY. When the compliance rate to in-person CBT changed from 75.7% to 92.1% (range for sensitivity analysis), the ICER of internet-based CBT increased from −20,254 USD/QALY to −10,117 USD/QALY. To further explore the interacting impact of the compliance to the two delivery forms of CBT, a two-way sensitivity analysis was performed on extended range (50‐100%) for both parameters. The findings of the two-way sensitivity analysis are shown in Figure 2. The gray area indicated the combination of compliance rates of internet-based and in-person CBT to prefer the internet-based CBT as the cost-effective option. The white area indicated the combinations of compliance rates leading to the in-person CBT as the cost-effective strategy.

The internet platform cost and percentage of psychotherapist’s time were previously reported to be major cost drivers of internet-based CBT [31,32]. The impact of these two parameters on the present base-case results were further evaluated by an extended sensitivity analysis, conducted by increasing the upper limit of internet platform cost from US $29 to US $2000, and the upper limit of psychotherapist’s time from 11.1% to 50%. The extended sensitivity analysis found a threshold value for the internet platform cost at US $1745, and no threshold was identified for psychotherapist’s time contribution.

**Figure 3.**
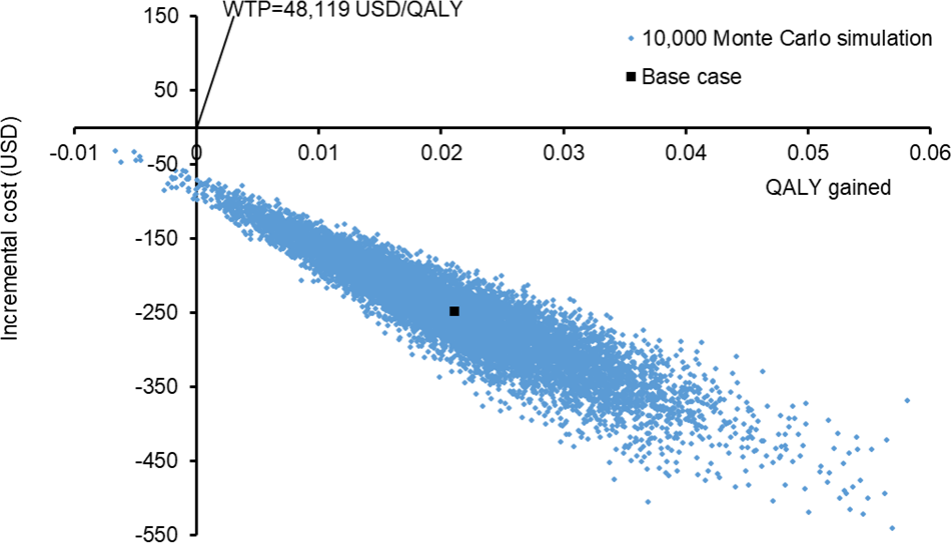
Scatter plot of increment cost and QALY saved by internet-based CBT group versus in-person CBT group. CBT: cognitive behavioral therapy; QALY: quality-adjusted life-year; WTP: willingness-to-pay.

The probabilistic sensitivity analysis recalculated the cost and QALYs for each study group 10,000 times using the Monte Carlo simulation. The incremental cost and additional QALYs gained by the internet group versus the in-person group in all Monte Carlo simulations are shown in Figure 3. The internet group was cost-saving in 100% of the 10,000 simulations, and gained higher QALYs 99.7% of the times (and therefore accepted as cost-effective in 99.7% of all simulations). The mean cost saved and additional QALY gained by the internet group were US $244 (95% CI US $242-246) and 0.0211 QALYs (95% CI 0.0209‐0.0213), respectively. The internet group remained the cost-effective option when the WTP was lowered from US $48,119/QALY to 0.

## Discussion

### Principal Results

This is the first study to report the cost-effectiveness of guided internet-based low-intensity CBT versus in-person CBT for mild depressive symptoms in university students. The present findings showed that the strategy of offering guided internet-based CBT gained higher QALYs and reduced school dropouts at a lower cost than the strategy of offering in-person CBT. The sensitivity analyses supported the robustness of these findings that the probability of the internet group to be accepted as cost-effective was 99.7% at the WTP threshold of US $48,119/QALY.

### Limitations

This study was limited by the nature of model-based analysis that the model structure only included the key clinical treatment outcomes of depressive symptoms. The model inputs were retrieved from multiple clinical studies with heterogeneity in the study subjects. The findings from studies of CBT for depressive symptoms in Hong Kong or Chinese population do not fully meet the needs of the present model, and some of the clinical model inputs were therefore obtained from findings published in Western populations [10-14,17-21]. The sources of model inputs might weaken the generalizability of the study results to the university students in Hong Kong. Also, the compliance to internet-based CBT was retrieved from a meta-analysis of studies on CBT among depressed adults, including students and older participants [14]. University students are likely to have a higher affinity (vs older) for internet-based therapy due to higher digital literacy [33], and the compliance estimate (including both student and older samples) might therefore have underestimated the compliance in students, thus the cost-effectiveness benefits of internet-based CBT in students. Sensitivity analysis was therefore conducted on all model inputs to examine the impact of model parameter variation on the model findings. The cost-effectiveness findings generated by the present model were found to be highly robust to the variation of all model inputs. The psychiatric outpatient cost estimation from a local cohort of university students did not differentiate the severity of depressive symptoms and might underestimate the total direct cost related to severe depression. Indirect cost (loss of productivity) and the quality-of-life change associated with depression-related university drop-out were not considered in the present model, and might therefore undervalue health economic benefits generated by the guided internet-based low-intensity CBT.

### Comparison With Prior Work

The individual compliance rates to the two CBT delivery modes were identified to be the most influential parameters in the one-way sensitivity analysis. The base-case values of CBT compliance were adopted from a meta-analysis on 24 studies comparing the adherence to guided internet-based CBT with the adherence to face-to-face CBT. Even though the average adherence of face-to-face CBT (83.9%) was higher than that of internet-based CBT (80.8%), the difference between adherence rates did not achieve statistical significance (*P*=.59) [14]. The impact of uncertainty in the difference between the compliance rates on the cost-effectiveness of the two CBT delivery modes was further elaborated by a two-way sensitivity analysis on the compliance rates of internet-based and in-person CBT. The findings of two-way sensitivity analysis provided information on the combinations of compliance rates required for internet-based and in-person CBT to be cost-effective. In cohort with low compliance to in-person CBT (<60%), the internet-based CBT was a cost-effective option if the compliance to internet-based CBT was at least 50%. In individuals with high compliance to in-person CBT (>90%), the internet-based CBT needed to achieve a compliance rate of >75% in order to be accepted as the cost-effective alternative. The CBT delivered by the internet-based technology was previously reported by a 6-month decision model in Australia to be less costly and more effective than “treatment as usual” (using antidepressant medication) for an acute depressive episode. Similar to the present findings, the compliance rate of internet-supported CBT was also identified as the most influential factor on the cost-effectiveness of the technology-based CBT [34].

A life-long model-based health economic analysis demonstrated that an internet-based CBT was more effective in gaining higher QALYs at higher cost than “treatment as usual” for depression in Spain, and the high cost (approximately US $500) of technology platform and recurring cost (approximately US $3000 per individual) were the major cost drivers of internet-based CBT [32]. A 3-year model-based health economic analysis in Germany reported that the internet-based CBT gained higher QALYs at a lower cost than in-person CBT for unipolar depression. The Germany study included a low-cost (US $53) technology platform and low staff cost (psychotherapist-time approximately 40% of in-person CBT) [31]. Similarly, the internet group in the present model adopted a low cost for the internet platform to deliver the low-intensity CBT materials (US $24) [25] and approximately 10% psychotherapist’s time of in-person CBT [26]. The cost-effectiveness of internet-based CBT was robust to high percentage (50%) of psychotherapist’s time, and it remained cost-effective if the internet platform cost was less than US $1745, as indicated by the findings of extended sensitivity analysis in the present study.

University students belong to a vulnerable group of depressive symptoms. The latest report of WHO showed the statistics of World Mental Health Surveys International College Student Project (n=13,984) that the prevalence of depressive episode was the highest of all common mental disorders. The lifetime prevalence of major depressive episode was 21.2% and the 12-month prevalence was 18.5% among the surveyed students [1]. CBT is the recommended intervention for mild depressive symptoms, yet the acceptance rate of professional treatment among young adults had been low (<30%) due to embarrassment and preferring to handle the issue alone [3,35]. The internet-based CBT, as a NICE recommended intervention, has been showed to enhance acceptance by university students for emotional needs [13] and therefore could meet the needs of university students with mild depressive symptoms. Our prior study on the cost-effectiveness of internet-supported CBT for mild symptoms of anxiety for university students reported that the internet-supported CBT gained higher QALYs with cost-saving from the societal perspective of Hong Kong [36]. The findings of the present study further indicated that offering internet-based CBT (versus in-person CBT) for mild depressive symptoms was effective in gaining higher QALYs and lowering dropout rate with cost-saving. The improvement in QALYs achieved statistical significance (as indicated in 10,000 Monte Carlo simulations), yet the improvement (by 0.0211 QALYs) was modest. The health economic benefits of guided internet-based low-intensity CBT were mainly generated by the cost-saving and similar effectiveness to in-person CBT. The internet-based low-intensity CBT therefore is an alternative for university students with mild depressive symptoms for the policy makers to consider, especially when manpower resources are limited and penetration of internet-based programs is adequate. To transfer the internet-based CBT cost-effectiveness demonstrated in the present study to university students in Hong Kong, cultural adaptation of the internet-based CBT is essential to address differences (that may influence the effectiveness and acceptability) in communication styles, stigma, and social values in the context of locality [37]. The effectiveness of culturally adapted internet-based CBT for Hong Kong adults (18‐70 years) with mild-to-moderate depressive symptoms has been recently demonstrated in a randomized clinical trial (n=402). The findings demonstrated a satisfactory recovery rate and supported that culturally and linguistically adapted internet-based CBT is an effective and feasible treatment for Hong Kong Chinese adults [38]. To further inform the decision-making process of resource allocation, future research to evaluate the culturally adapted internet-based CBT for university students with mild depressive symptoms together with feasibility study are highly warranted.

### Conclusions

The guided internet-based low-intensity CBT was found to gain higher QALYs and reduce dropout rate at a lower cost, when compared to in-person CBT, for mild depressive symptoms in university students from the perspective of service providers in Hong Kong.

## Abbreviations

CBT: cognitive behavioral therapy
GDP: gross domestic product
ICER: incremental cost per quality-adjusted life year
NICE: National Institute for Health and Care Excellence
PHQ-9: Patient Health Questionnaire-9
QALY: quality-adjusted life year
WHO: World Health Organization
WTP: willingness-to-pay
